# TAK1: a potent tumour necrosis factor inhibitor for the treatment of inflammatory diseases

**DOI:** 10.1098/rsob.200099

**Published:** 2020-09-02

**Authors:** Juliane Totzke, Scott A. Scarneo, Kelly W. Yang, Timothy A. J. Haystead

**Affiliations:** Department of Pharmacology and Cancer Biology, Duke University School of Medicine, Durham, NC 27710, USA

**Keywords:** TAK1, TNF, inflammation, kinase, rheumatoid arthritis

## Abstract

Aberrant tumour necrosis factor (TNF) signalling is a hallmark of many inflammatory diseases including rheumatoid arthritis (RA), irritable bowel disease and lupus. Maladaptive TNF signalling can lead to hyper active downstream nuclear factor (NF)-κβ signalling in turn amplifying a cell's inflammatory response and exacerbating disease. Within the TNF intracellular inflammatory signalling cascade, transforming growth factor-β-activated kinase 1 (TAK1) has been shown to play a critical role in mediating signal transduction and downstream NF-κβ activation. Owing to its role in TNF inflammatory signalling, TAK1 has become a potential therapeutic target for the treatment of inflammatory diseases such as RA. This review highlights the current development of targeting the TNF-TAK1 signalling axis as a novel therapeutic strategy for the treatment of inflammatory diseases.

## 1. Introduction

Tumour necrosis factor (TNF) is a pleotropic proinflammatory cytokine widely regarded as the master regulator of proinflammatory signalling. Its role in mediating inflammatory diseases, such as autoimmune and chronic pain syndromes, has been widely documented and resulted in the development of targeted therapies aimed at inhibiting TNF [1]. To date, anti-TNF targeted biologics, such as etanercept and adalimumab, represent a 25 billion dollar industry with the majority of use in the treatment of rheumatoid arthritis (RA) and other auto-immune indications [2]. In these diseases, TNF has been shown to play an integral role in the pathogenesis of aberrant maladaptive inflammatory signalling in the absence of physiological needs. This elevated TNF signalling activates immune cells in the joints of patients, leading to increased infiltration of immune cells, resulting in the deterioration of bone and synovial tissue and causing chronic joint pain and destruction. Anti-TNF-based therapies decrease TNF concentration and function, therefore dampening proinflammatory signalling mediated by nuclear factor (NF)-κβ, p38 and c-JUN N-terminal kinase (JNK). Despite significant attenuation of disease in many RA patients, the development of antibodies against biological anti-TNF therapies can limit their long term use, resulting in up to 40% of patients failing to realize long-term disease mitigation [3]. Additionally, anti-TNF biological therapies completely remove all circulating TNF, potentially limiting critical immune-pathogen detection leading to increased risk of infection in patients [4]. By contrast, small molecule inhibitors of TNF expression can be dialled in to reduce TNF expression without complete TNF signalling ablation, allowing critical immune-pathogen detection to occur and potentially reducing the increased risk associated with biological therapeutics. Therefore, the development of small-molecule inhibitors which circumnavigate issues observed in biological anti-TNF therapies may provide a novel treatment strategy for TNF mediated inflammatory diseases.

## 2. TAK1

**Figure 1.**
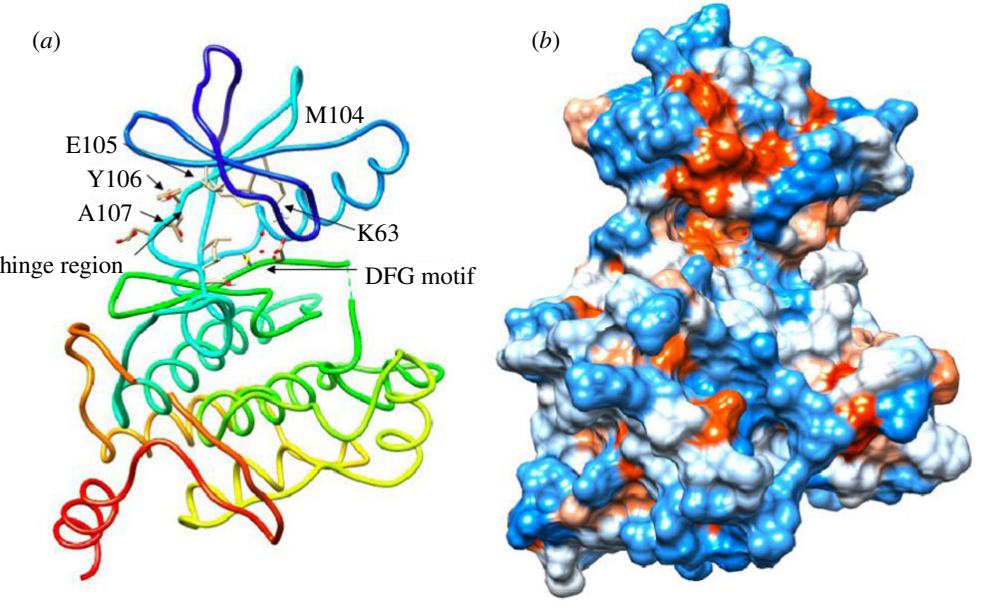
Crystal structure of TAK1 (PDB:5V5N); ribbon (*a*), hydrophobicity surface (*b*). Key interacting residues highlighted. Binding surface illustrates hydrophobicity surface, hydrophilic (blue) and hydrophobic (red) regions of TAK1 ATP binding pocket.

Transforming growth factor-β-activated kinase 1 (TAK1) serves as a key node in the TNF inflammatory signalling pathway. It is a serine/threonine kinase and can be activated by a range of proinflammatory cytokines and ligands including TNF, interleukin-1β (IL-1β), liposaccharide (LPS) and transforming growth factor beta (TGFβ) [5]. TAK1 is an evolutionarily conserved member of the MAP3 K family and cluster with the tyrosine-like and sterile kinase families [6]. Similar to the majority of kinases, TAK1 contains an N (residues 1–104)- and C (residues 111–303)-terminus connected through the hinge region (Met 104-Ser 111). This region provides an opening for the ATP binding pocket. Additionally, like most kinases, TAK1 has a catalytic lysine in the active site (Lys 63, figure 1). The purine moiety of ATP forms two hydrogen bonds with residues Ala 107 and Glu 105 [7]. Crystallization of inactive adenosine-bound TAK1 demonstrated a critical hydrogen bond of the ribose 3′-O to Pro 160. Further hydrogen bonding is observed to Asp 175, which is the leading residue of the DFG motif. This residue is thought to interact with Lys 63 through polar interactions and is catalytically important for phosphate transfer to substrate molecules [7]. Critical for TAK1-TGF-beta-activated kinase 1 (TAB1) interactions is a helical loop centred around Phe 484, which provides extensive surface contact between the two proteins. A similar protein interaction has been observed during the functional activation of c-Abl; the myristoylated N-terminus of c-Abl binds to the base of its C-terminus and allows a conformational change to incorporate SRC homology (SH)2 and SH3 domain binding [8]. Because the interacting protein binds in close proximity to the helix aI in both TAK1 and c-Abl, it can be speculated that a conformational change in the TAK1 C-terminus occurs to fully incorporate TAB1, which is part of a more complex kinase activation process. Based on the importance of TAB1 during the TAK1 activation process, most enzymatic studies of TAK1 use a TAK1-TAB1 fusion protein to assess functionalities of the activated kinase. In addition to enzymatic studies, *in vitro* experiments using cytokine stimulations have shown that TAK1 forms a ternary complex with TAB1 and TAB2/3 and undergoes phosphorylation on residues of its activation loop (Thr 178, Thr 184, Thr 187, Ser 192) and K63-linked polyubiquitination at Lys 158 to be fully functional [9,10]. While polyubiquitination of TAK1 is required for signal transduction in cells, kinase activity can be modelled by TAK1-TAB1 fusion proteins for kinetic and enzymatic studies [11].

## 3. TAK1 and cytokine signalling

### 3.1. TAK1–tumour necrosis factor signalling

**Figure 2.**
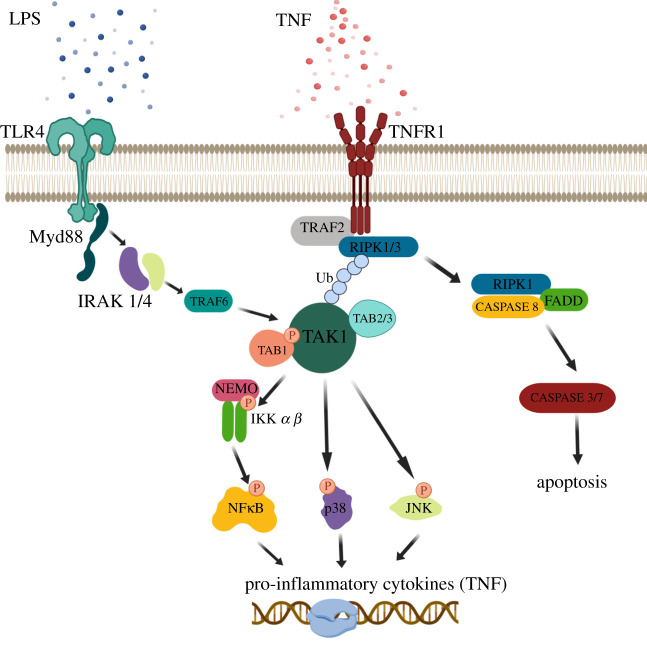
TAK1 inflammatory signalling pathways. TAK1 is activated by many inflammatory signalling ligands, such as TNF and LPS which bind TNF-receptor 1 (TNFR1) and toll-like receptor (TLR4). Following upstream ligand stimulation, TAK1 complexes with its adapter proteins TAK-binding protein 1 (TAB1) as well as TAB2 and 3. TAK1 phosphorylation leads to activation of downstream inflammatory signalling including nuclear factor (NF-κβ), p38 and JNK.

Much of the intracellular actions of TNF are thought to be mediated through TAK1 (figure 2). Following binding of TNF to the TNF-Receptor 1 (TNFR1), TAK1 in complex with TAB1–3 activates inhibitor of nuclear factor kappa-B kinase (IKK), leading to ubiquitination and proteasomal degradation of IKβ subunit subsequently leading to activation and translocation of NF-κβ to the nucleus for TNF gene transcription [12]. In addition to NF-κβ signalling, TAK1 can also signal through MKK3/4/6, which leads to p38 and JNK activation and subsequent translocation to the nucleus to activate transcription of pro-survival and proinflammatory cytokine genes [12,13] (figure 2). Receptor interacting serine/threonine kinase 1 (RIPK1) is another parallel regulator in TNF-mediated cell death. While RIPK1 activation can trigger its own apoptosis pathways, TAK1 inhibition, regardless of subsequent NF-κβ signalling, can also promote TNF-mediated RIPK1-dependent cell death [14,15].

In addition to blocking intracellular TNF signalling through the TNF receptor, TAK1 inhibition has also been shown to downregulate TNF expression from other inflammatory ligands such as LPS [12]. Data have shown that TAK1 inhibition with the TAK1 inhibitor, takinib, leads to potent inhibition of TNF secretion and signalling in immune cells. Previous work by Scarneo *et al*. showed following stimulation of THP-1 macrophages by LPS and interferon gamma (IFN*γ*), cells treated with the TAK1 inhibitor, takinib, showed selective inhibition of TNF (greater than ninefold reduction) compared to 110 other cytokines/chemokines profiled. Interestingly, other inflammatory cytokines stimulated by LPS and IFN*γ*, such as interleukin-6 (IL-6) and IL-1β, showed significantly less suppression by TAK1 inhibition, supporting the role that TAK1 signalling potently regulates TNF expression over other inflammatory cytokines [12]. Further evidence supporting TAK1's role in TNF signalling comes from *in vitro* genetic knockout studies in THP-1 macrophages. Genetic knockout of TAK1 has shown that loss of TAK1 blocks LPS induced TNF pro-inflammatory responses [12]. Thus, it has become apparent that TAK1 potently regulates TNF signalling in immune cells.

### 3.2. TAK1-interleukin-1β signalling

In addition to its role in TNF signalling, TAK1 regulates various upstream inflammatory signalling cascades, including the inflammatory cytokine IL-1β. Like TNF, IL-1β signalling regulates a variety of inflammatory responses following infection. Many immune cells (i.e. neutrophils, and Langerhans cells) and epithelial cells are sensitive to IL-1β, which can induce transmigration to target tissues including cartilage, bone, blood vessels, the hypothalamus and the pancreas [16,17]. Following IL-1β binding to its receptor (IL-1-R1), a high-affinity multimeric complex consisting of IL-1, IL-1-R1 and IL-1 receptor-accessory protein (IL-1-RAP) is formed [18]. This complex recruits MyD88, IL-1 receptor-associated kinase 1 (IRAK1) and IL-1 receptor-associated kinase 4 (IRAK4). IRAK1 and IRAK4 then associate with TRAF6 and recruit TAB2 to the complex. TNF receptor associated factor (TRAF)6 is an E3-ubiquitin ligase and post-translationally modifies TAB2 through K63-polyubiquitination. Formation of the TRAF6-TAB2-TAK1 complex is necessary for TAK1 activation. In addition to TAK1's role in regulating downstream IL-1β signalling, inhibition of TAK1 has shown to modestly reduce IL-1β secretion in stimulated macrophages [12].

### 3.3. TAK1-toll-like receptor signalling

Toll-like receptors (TLRs) are a large receptor family and belong to the pattern recognition receptors (PRR), which are a crucial part of innate inflammation and immunity. While several endogenous and exogenous ligands for TLRs are known, TLR4 will be discussed because of the importance of its LPS ligand and downstream TAK1 signalling. Following LPS binding to TLR4, a signalling cascade similar to IL-1β is activated. MyD88 recruits IRAK proteins, leading to TAK1 activation [19] (figure 2). TAK1 has been shown to play a critical role in mediating LPS and TLR4 inflammatory signalling cascades. For example, pharmacological inhibition of TAK1 has been shown to block both *in vitro* and *in vivo* immune response to LPS [20–22]. In the LPS sepsis mouse model, previous work has shown that administration of takinib blocked LPS induced increases in serum TNF [12].

### 3.4. TAK1-transforming growth factor β signalling

TGFβ is a pleiotropic cytokine that regulates angiogenesis, migration, cell proliferation, differentiation and apoptosis in a variety of tissues [23]. TGFβ binds to TGFβ-RII (TGFβ type II receptor) and induces dimerization of receptor monomers. TGFβ-RII recruits and phosphorylates two TGFβ-RI (TGFβ type I receptor) molecules with its cytoplasmic kinase domain, subsequently leading to Smad phosphorylation. The Smad-dependent signalling pathway results in transcription of genes involved in apoptosis, immune suppression, and extracellular matrix (ECM) neogenesis [24]. Recent studies suggest that TAK1 activation in TGFβ signalling functions in a receptor kinase-independent manner [25,26]. TRAF6 associates with TGFβ26 R1 following TGFβ binding, which leads to polyubiquitination of TAK1 and NF-κβ, p38, and JNK activation, which subsequently triggers a proliferative and inflammatory transcriptional programme. Although TGFβ was originally thought to be a major activator of TAK1, growing consensus in the field recognizes TGFβ as a weak activating ligand in the TAK1 pathway.

## 4. TAK1 as a target for inflammatory disorders

Inflammatory disorders, such as RA, Crohn's disease and inflammatory bowel disease (IBD), have shown increased expression of TNF, IL-1β and IL-6, which contribute to joint erosion and tissue destruction [27–30]. Genome-wide association studies (GWAS) identified several disease-relevant loci related to TAK1-TNF signalling, which primes TAK1 as a therapeutic target. For example, GWAS meta-analysis in RA patients from Asia found enrichment of *MAP3K7* (TAK1) as well as enrichment of the TAK1 activator protein TAB1 [31]. Furthermore, single nucleotide polymorphisms (SNPs) in the TAK1-TNF pathway including *MAP3K7* and TNFSF18 (Crohn's disease), TNFSF14 and 16NFKB1 (ulcerative colitis), TRAF3IP2 and TNFAIP2 (general inflammatory bowel disorder) are associated with higher risk for disease development [32–34]. Although many of these SNP's have been identified to influence one's risk towards disease development, further studies are needed to tease out the functional implications of the individual SNPs on protein function. Table 1 provides an overview of diseases for which TAK1 has been implicated as a potential drug target.

**Table 1.** Inflammatory disorders with TAK1 indication.

inflammatory disorders	references
rheumatoid arthritis	[35,36]
inflammatory bowel syndrome	[37]
kidney disease	[38,39]
skin inflammation	[40–42]
chronic pain	[43,44]

In RA, TNF is overexpressed and promotes disease progression. Following initial joint injury, immune cells secrete TNF, leading to a tumour-like cell transformation of fibroblast-like synoviocytes (FLS) and increased migration, protease expression, and secretion of inflammatory mediators [45]. While effective biological therapeutics targeting TNF and TNF-R have been established in recent years, non-responders and cases of resistance have been reported [46,47]. With the arrival of selective TAK1 inhibitors, pre-clinical pharmacological studies have established the therapeutic potential of TAK1 inhibition in RA. For example, our group found that daily administration of takinib, a TAK1 inhibitor, significantly reduced the clinical arthritic score of mice in the collagen-induced arthritis (CIA) mouse model of human RA. In addition, we found that inhibition of TAK1 in human-derived RA-FLS cells leads to decreased activation of intracellular NF-κβ signalling as well as a reduction in inflammatory cytokine expression [36].

### 4.1. *In vivo* genetic evidence of TAK1

Based on the potent regulation of TNF signalling in immune cells, TAK1 may represent a novel intracellular therapy for TNF mediated diseases. However, *in vivo* genetic studies in mice have raised doubts to the therapeutic potential of TAK1. Early studies of TAK1 *in vivo* had shown that global knockout of TAK1 in mice led to early embryonic lethality owing to abnormal neural tube development [10,48]. Additionally, knockout of co-stimulatory proteins TAB1 and 2, which are necessary for TAK1 activation, are not viable owing to cardiovascular and liver abnormalities, respectively [49,50]. Thus, the use of conditional knockouts of TAK1 using tissue-specific promotors have provided greater insight of the *in vivo* function of TAK1. Immune cell-specific knockouts such as B cell-TAK1^KO^ demonstrated impaired B cell maturation and antigen-induced immune responses [48]. Non-immune cell conditional knockout studies have been performed in the epidermis, liver parenchyma, and enterocytes and have resulted in abnormal cell development and inflammatory phenotypes [51–53].

Loss of function TAK1 mutations in humans have not yet been identified, but recent discovery of a gain of function developmental phenotype owing to expression of a truncated form of the protein kinase has been reported [54]. Patients with this TAK1 gain of function mutation present with craniofacial abnormalities [54,55]. These deficits reflect the actions of an unregulated TAK1 protein kinase during embryogenesis leading to severe developmental issues. As with many other kinase drug targets, often genetic data has contradicted pharmacological studies, complicating their therapeutic potential [56,57]. This is largely because many protein kinase-mediated pathways either have built in redundancy, or the cell compensates by reprograming its signalling networks [58]. Additionally, protein kinases like TAK1 have scaffolding functions in addition to its enzymatic functions [59]. Removal of the protein therefore is likely to have impacts on other pathways, which makes interpretation of genetic *in vivo* studies of TAK1 difficult. Despite contradictory genetic data supporting the therapeutic potential of TAK1, early pharmacological data have strongly supported the potential of pharmacological manipulation as a therapeutic approach. Benefits of a pharmacological approach include the retention of TAK1's scaffolding function as well as ability to restore TNF signalling to normal levels rather than completely ablate it. Thus, the development of a selective TAK1 inhibitor will advance our understanding of the therapeutic potential of TAK1 in disease contexts.

### 4.2. Pharmacological development of TAK1 inhibitors

**Figure 3.**
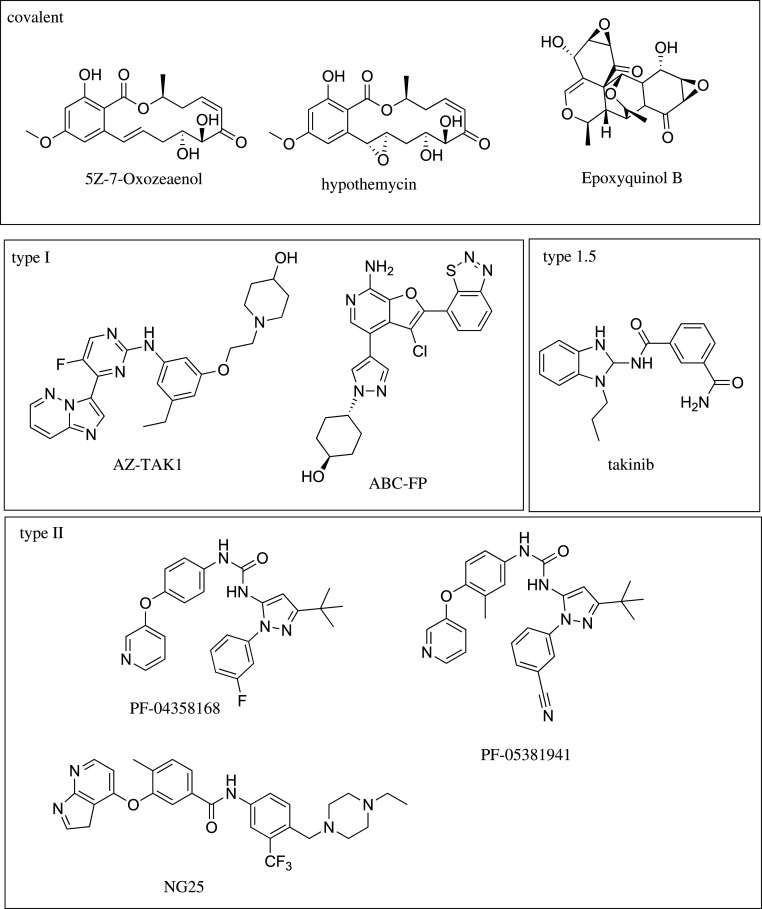
Chemical structures of current TAK1 inhibitors by covalent, type I, type 1.5 and type II binding mode.

Owing to its role in mediating TNF signalling, TAK1 is a potential novel drug target for the treatment of inflammatory diseases. Until recently, pharmacological inhibitors of TAK1 lacked selectivity in the kinome, limiting their use to support the therapeutic role of TAK1 in diseases. However, discovery of the selective TAK1 inhibitor, takinib, has provided the first selective chemical entity to probe the therapeutic potential of TAK1 in the absence of off target inhibition [13]. Previously, covalent, type I and type II kinase inhibitors for TAK1 have been identified (table 2 and figure 3). Type I inhibitors target the kinase in the active DFG-in conformation, in which the DFG aspartate residue is pointed into the ATP binding site. In a DFG-out conformation, the aspartate and phenylalanine residues switch positions, creating an allosteric pocket next to the ATP binding site. While ATP binding is now sterically blocked, type II inhibitors target the kinase in this inactive DFG-out conformation. The most widely used tool to study TAK1 biology is the resorcylic lactone (5Z)-7-Oxozeaenol (5ZO) [60]. 5ZO demonstrated increased efficacy compared to the Federal Drug Administration-approved Janus kinase inhibitor tofacitinib in RA cell models [35]. However, this molecule also potently inhibits a panel of at least 50 other kinases (RIOK3, MEK1,2,3,4,5 PDGFRB, FLT4, FLT1/3, KIT, TGFBR2) and forms a covalent bond with reactive cysteines in the activation loop of its targets, rendering it inadequate for therapeutic purposes owing to off target effects [60,69]. Despite the known limitations of 5ZO, it is still widely used to study TAK1 biology in contexts of disease. Structurally related compounds of 5ZO suffer from similar selectivity issues; for example, hypothemycin also targets mitogen-activated kinase kinase (MEK) (IC_50_ 15 nM) and suppresses IL-2 production (IC_50_ 9 nM) [61,70,71]. Progress has been made with type I hinge region binders to the ATP binding pocket of TAK1. AZ-TAK1 has shown efficacy in acute myeloid leukemia, but it lacked selectivity characterization in a large kinase panel [72]. In an initial study, AZ-TAK1 inhibited TAK1 with an IC_50_ < 100 nM, but also showed low nM potency against HIPK2 (IC_50_ 3 nM) and CDK9 (IC_50_ 9 nM) [63]. Another type I binder, ABC-FP, also lacks selectivity profiling. NG-25 was recently developed and targets TAK1 in the DFG-out conformation (type II binder). As a dual inhibitor, it targets TAK1 and MAP4K2 (IC_50_ 22 nM) with similar potencies [67]. Drug companies have developed several other small-molecule inhibitors with diverse chemotypes, but little information is currently available on these compounds including the widely used LYTAK1 which has been removed from the public domain [61]. Recently, the discovery of the takinib scaffold has provided the first selective and potent (IC_50_ 9 nM) TAK1 inhibitor [13]. In a panel of 146 human kinases, takinib showed a 12-fold degree of selectivity over the next kinase, IRAK4. The development and characterization of takinib has provided the first selective pharmacological tool to study TAK1's therapeutic potential [13,36].

**Table 2.** TAK1 affinity of current TAK1 inhibitors.

name	IC_50_ [nM]	PDB crystal structure	binding mode	reference
5Z-7-Oxozeaenol	9	4GS6	covalent	[60]
hypothemycin	100	n.a.	covalent	[61]
epoxyquinol	20000	n.a.	covalent	[62]
AZ-TAK1	8	unpublished	Type I	[63]
ABC-FP	28	4L53	Type I	[64,65]
PF-04358168	1640	2YIY	Type II	[66]
PF-05381941	156	N/A	Type II	[66]
NG-25	4	4O91	Type II	[67]
LYTAK1	N/A	n.a.	N/A	[68]
takinib	9.5	5V5N	Type 1.5	[13]

The limited development of potent TAK1 inhibitors may be a result of targeting the kinase in the active/DFG-in/type I or inactive/DFG-out/type II conformation. While type I binders often form hydrogen bonds within the hinge region of the ATP binding site, type II binders extend beyond the hinge region into a hydrophobic pocket in close proximity to the ATP-binding site and are generally of higher molecular weight. A general core structure for type II binders has been developed, consisting of a hydrophobic moiety with hydrogen-bond acceptors, a linker region that often consists of amide moieties, and another hydrophobic moiety with hydrogen bond donors and acceptors [67,73]. It is not clear if every kinase readily undergoes conformational changes from the DFG-in to DFG-out conformation (less than 10% of kinases in the protein databank are in the DFG-out conformation). The relative conformational flexibility of TAK1 is underlined in the fact that the activation loop of TAK1 has not been crystalized. Structural disorder of this region is often cited as a reason for the missing residues of the activation loop in TAK1 crystal structures. While the DFG-in conformation is conserved across kinases, the DFG-out conformation is unique to each kinase, potentially allowing for more selective inhibitors. However, the higher selectivity of type II inhibitors could also stem from the fact that less kinases adopt this conformation and therefore a smaller sample size is probed for these inhibitors.

**Figure 4.**
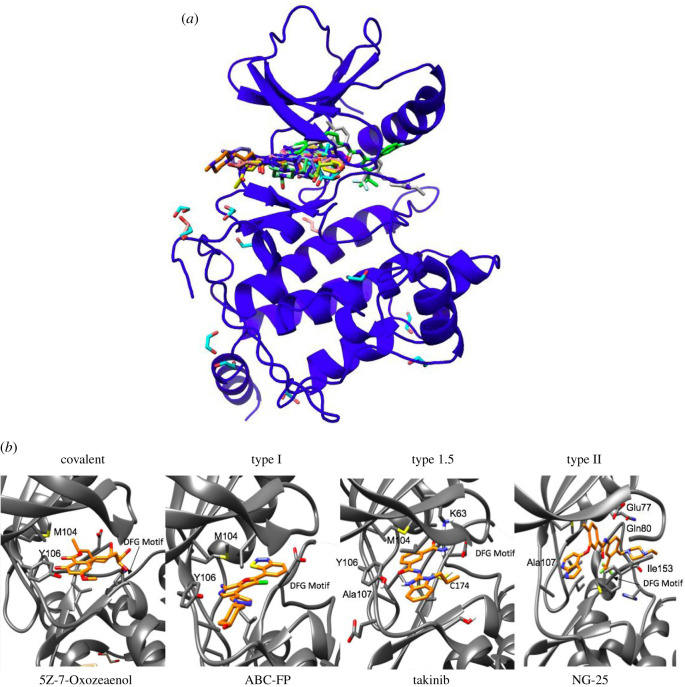
TAK1 inhibitors in the ATP binding pocket of TAK1. (*a*) Overlay of covalent, type I, and type II inhibitors in the TAK1 ATP binding pocket. (*b*) Key interacting residues of the covalent TAK1 inhibitor 5Z-7-Oxozeanol (PDB 4GS6), type I ABC-FP (PDB 4L53), type 1.5 takinib (PDB 5V5N) and type II NG-25 (PDB 4O91).

All previously described TAK1 inhibitors occupy the ATP-binding pocket of TAK1, and no allosteric binders have been described to this date (figure 4). From the overlay of TAK1 inhibitors, it can be observed that the type II binders NG-25 and PF-04358168 ((grey, PDB 4O91), (green, PDB 2YIY)) span across the ATP-binding pocket from the hinge region to the DFG motif and into a small hydrophobic binding pocket that becomes available in the DFG-out conformation. Both compounds hydrogen-bond to amino acid residues critical for kinase function: Ala 107 (hinge region), Glu 77 (aC loop), 23 and Asp 175 (DFG motif). 5ZO (turquoise, PDB 4GS6) forms a covalent bond with Cys 174 and hydrogen-bonds to Ala 107 and Pro 160. Ligand 10 (purple, PDB 4JGA), 11 (orange, PDB 4JGB) and 12 (dark green, PDB 4JGD) are type I binders derived from a series of lead optimizations [74]. They interact with Ala 107, Asp 175 and Ser 111. Ser 111 interactions had not been previously described for other TAK1 inhibitors. Optimized 7-aminofuro[2,3- c]pyridine behaves as classical hinge region binders ((pink PDB 4L3P), (yellow, PDB 4L52), (salmon, PDB 4L53)) and interact with Ala 107 and Glu 105. At present, TAK1 inhibitors share commonalities in key interaction residues observed for covalent, type I, and type II inhibitors.

## 5. Conclusion

TAK1 represents a novel drug target for the regulation of TNF signalling in autoimmune diseases. As a key node in major disease-related cytokine signalling pathways, it activates proliferative and inflammatory gene programmes relevant to disease progression. Despite its biological importance, a lack of selective and potent TAK1 inhibitors and contradictory genetic evidence had greatly reduced our therapeutic understanding of TAK1. The most widely used TAK1 inhibitor 5ZO is a nonselective natural product, which inherently limits conclusions of studies with this compound. However, recent advent of novel TAK1 inhibitors such as takinib have revitalized the field and provided the first selective TAK1 inhibitor, allowing for enhanced understanding and evaluation of the therapeutic potential of TAK1 in inflammatory disorders.

## Data accessibility

All data generated in this review can be shared by contacting Timothy Haytsead (timothy.haystead@duke.edu).

## References

[1] Feldmann M 2002 Development of anti-TNF therapy for rheumatoid arthritis. Nat. Rev. Immunol. 2, 364–371. (10.1038/nri802) 12033742

[2] Monaco C, Nanchahal J, Taylor P, Feldmann M 2015 Anti-TNF therapy: past, present and future. Int. Immunol. 27, 55–62. (10.1093/intimm/dxu102) 25411043 PMC4279876

[3] Li P, Zheng Y, Chen X 2017 Drugs for autoimmune inflammatory diseases: from small molecule compounds to anti-TNF biologics. Front. Pharmacol. 8, 460 (10.3389/fphar.2017.00460) 28785220 PMC5506195

[4] Yrjanainen H, Hytönen J, Song XY, Oksi J, Hartiala K, Viljanen MK. 2007 Anti-tumor necrosis factor-alpha treatment activates *Borrelia burgdorferi* spirochetes 4 weeks after ceftriaxone treatment in C3H/He mice. J. Infect. Dis. 195, 1489–1496. (10.1086/513873) 17436229

[5] Sakurai H 2012 Targeting of TAK1 in inflammatory disorders and cancer. Trends Pharmacol. Sci. 33, 522–530. (10.1016/j.tips.2012.06.007) 22795313

[6] Manning G, Whyte DB, Martinez R, Hunter T, Sudarsanam S 2002 The protein kinase complement of the human genome. Science 298, 1912–1934. (10.1126/science.1075762) 12471243

[7] Brown K, Vial SCM, Dedi N, Long JM, Dunster NJ, Cheetham GMT 2005 Structural basis for the interaction of TAK1 kinase with its activating protein TAB1. J. Mol. Biol. 354, 1013–1020. (10.1016/j.jmb.2005.09.098) 16289117

[8] Nagar B, Hantschel O, Young MA, Scheffzek K, Veach D, Bornmann W, Clarkson B, Superti-Furga G, Kuriyan J 2003 Structural basis for the autoinhibition of c-Abl tyrosine kinase. Cell 112, 859–871. (10.1016/S0092-8674(03)00194-6) 12654251

[9] Wang C, Deng L, Hong M, Akkaraju GR, Inoue J-, Chen ZJ 2001 TAK1 is a ubiquitin-dependent kinase of MKK and IKK. Nature 412, 346–351. (10.1038/35085597) 11460167

[10] Shim JH et al. 2005 TAK1, but not TAB1 or TAB2, plays an essential role in multiple signaling pathways *in vivo*. Genes Dev. 19, 2668–2681. (10.1101/gad.1360605) 16260493 PMC1283960

[11] Sakurai H, Nishi A, Sato N, Mizukami J, Miyoshi H, Sugita T,. 2002 TAK1-TAB1 fusion protein: a novel constitutively active mitogen-activated protein kinase kinase kinase that stimulates AP-1 and NF-κB signaling pathways. Biochem. Biophys. Res. Commun. 297, 1277–1281. (10.1016/S0006-291X(02)02379-3) 12372426

[12] Scarneo SA et al. 2018 Genetic and pharmacological validation of TAK1 inhibition in macrophages as a therapeutic strategy to effectively inhibit TNF secretion. Sci. Rep. 8, 17058 (10.1038/s41598-018-35189-7) 30451876 PMC6242965

[13] Totzke J et al. 2017 Takinib, a selective TAK1 inhibitor, broadens the therapeutic efficacy of TNF-alpha inhibition for cancer and autoimmune disease. Cell Chem. Biol. 24, 1029–1039 e1027. (10.1016/j.chembiol.2017.07.011) 28820959 PMC5576570

[14] Geng J et al. 2017 Regulation of RIPK1 activation by TAK1-mediated phosphorylation dictates apoptosis and necroptosis. Nat. Commun. 8, 359 (10.1038/s41467-017-00406-w) 28842570 PMC5572456

[15] Scarneo SA, Yang KW, Roques JR, Dai A, Eibschutz LS, Hughes P, Haystead TAJ 2020 TAK1 regulates the tumor microenvironment through inflammatory, angiogenetic and apoptotic signaling cascades. Oncotarget 11, 1961–1970. (10.18632/oncotarget.27606) 32523651 PMC7260121

[16] Cumberbatch M, Dearman RJ, Kimber I 1997 Langerhans cells require signals from both tumour necrosis factor-alpha and interleukin-1β for migration. Immunology 92, 388–395. (10.1046/j.1365-2567.1997.00360.x) 9486113 PMC1363801

[17] Oliveira SH, Canetti C, Ribeiro RA, Cunha FQ 2008 Neutrophil migration induced by IL-1β depends upon LTB4 released by macrophages and upon TNF-α and IL-1β released by mast cells. Inflammation 31, 36–46. (10.1007/s10753-007-9047-x) 17874178

[18] Qian Y, Commane M, Ninomiya-Tsuji J, Matsumoto K, Li X 2001 IRAK-mediated translocation of TRAF6 and TAB2 in the interleukin-1-induced activation of NF-κB. J. Biol. Chem. 276, 41 661–41 667. (10.1074/jbc.M102262200) 11518704

[19] Hornef MW, Normark BH, Vandewalle A, Normark S 2003 Intracellular recognition of lipopolysaccharide by toll-like receptor 4 in intestinal epithelial cells. J. Exp. Med. 198, 1225–1235. (10.1084/jem.20022194) 14568981 PMC2194240

[20] Wang X, Wang C, Wang J, Zhao S, Zhang K, Wang J, Zhang W, Wu C, Yang J 2014 Pseudoginsenoside-F11 (PF11) exerts anti-neuroinflammatory effects on LPS-activated microglial cells by inhibiting TLR4-mediated TAK1/IKK/NF-κB, MAPKs and Akt signaling pathways. Neuropharmacology 79, 642–656. (10.1016/j.neuropharm.2014.01.022) 24467851

[21] Wang W, Xia T, Yu X 2015 Wogonin suppresses inflammatory response and maintains intestinal barrier function via TLR4-MyD88-TAK1-mediated NF-κB pathway *in vitro*. Inflamm. Res. 64, 423–431. (10.1007/s00011-015-0822-0) 25917044

[22] Li X, Lian L-H, Bai T, Wu Y-L, Wan Y, Xie W-X, Jin X, Nan J-X 2011 Cryptotanshinone inhibits LPS-induced proinflammatory mediators via TLR4 and TAK1 signaling pathway. Int. Immunopharmacol. 11, 1871–1876. (10.1016/j.intimp.2011.07.018) 21835267

[23] Sporn MB, Roberts AB 1990 TGF-beta: problems and prospects. Cell Regul. 1, 875–882. (10.1091/mbc.1.12.875) 2100192 PMC362858

[24] Wrana JL, Attisano L, Cárcamo J, Zentella A, Doody J, Laiho M, Wang XF, Massague J 1992 TGF beta signals through a heteromeric protein kinase receptor complex. Cell 71, 1003–1014. (10.1016/0092-8674(92)90395-S) 1333888

[25] Sorrentino A, Thakur N, Grimsby S, Marcusson A, Von Bulow V, Schuster N, Zhang S, Heldin CH, Landström M 2008 The type I TGF-β receptor engages TRAF6 to activate TAK1 in a receptor kinase-independent manner. Nat. Cell. Biol. 10, 1199–1207. (10.1038/ncb1780) 18758450

[26] Gudey SK, Sundar R, Mu Y, Wallenius A, Zang G, Bergh A, Heldin C-H, Landstrom M 2014 TRAF6 stimulates the tumor-promoting effects of TGFβ type I receptor through polyubiquitination and activation of presenilin 1. Sci. Signal 7, ra2 (10.1126/scisignal.2004207) 24399296

[27] Matsuno H et al. 2002 The role of TNF-alpha in the pathogenesis of inflammation and joint destruction in rheumatoid arthritis (RA): a study using a human RA/SCID mouse chimera. Rheumatology (Oxford) 41, 329–337. (10.1093/rheumatology/41.3.329) 11934972

[28] Wooley PH, Whalen JD, Chapman DL, Berger AE, Richard KA, Aspar DG, Staite ND 1993 The effect of an interleukin-1 receptor antagonist protein on type II collagen-induced arthritis and antigen-induced arthritis in mice. Arthritis Rheumatol. 36, 1305–1314. (10.1002/art.1780360915) 8216424

[29] Geiger T, Towbin H, Cosenti-Vargas A, Zingel O, Arnold J, Rordorf C, Glatt M, Vosbeck K 1993 Neutralization of interleukin-1 beta activity *in vivo* with a monoclonal antibody alleviates collagen-induced arthritis in DBA/1 mice and prevents the associated acute-phase response. Clin. Exp. Rheumatol. 11, 515–522. 8275587

[30] Ishihara K, Hirano T 2002 IL-6 in autoimmune disease and chronic inflammatory proliferative disease. Cytokine Growth Factor Rev. 13, 357–368. (10.1016/S1359-6101(02)00027-8) 12220549

[31] Okada Y, Raj T, Yamamoto K 2016 Ethnically shared and heterogeneous impacts of molecular pathways suggested by the genome-wide meta-analysis of rheumatoid arthritis. Rheumatology (Oxford) 55, 186–189. (10.1093/rheumatology/kev314) 26320133

[32] Eleftherohorinou H, Wright V, Hoggart C, Hartikainen A-L, Jarvelin M-R, Balding D, Coin L, Levin M 2009 Pathway analysis of GWAS provides new insights into genetic susceptibility to 3 inflammatory diseases. PLoS ONE 4, e8068 (10.1371/journal.pone.0008068) 19956648 PMC2778995

[33] Fuyuno Y et al. 2016 Genetic characteristics of inflammatory bowel disease in a Japanese population. J. Gastroenterol. 51, 672–681. (10.1007/s00535-015-1135-3) 26511940

[34] Jostins L et al. 2012 Host-microbe interactions have shaped the genetic architecture of inflammatory bowel disease. Nature 491, 119–124. (10.1038/nature11582) 23128233 PMC3491803

[35] Jones DS, Jenney AP, Swantek JL, Burke JM, Lauffenburger DA, Sorger PK 2017 Profiling drugs for rheumatoid arthritis that inhibit synovial fibroblast activation. Nat. Chem. Biol. 13, 38–45. (10.1038/nchembio.2211) 27820799 PMC5372219

[36] Scarneo SA, Eibschutz LS, Bendele PJ, Yang KW, Totzke J, Hughes P, Fox DA, Haystead TA 2019 Pharmacological inhibition of TAK1, with the selective inhibitor takinib, alleviates clinical manifestation of arthritis in CIA mice. Arthritis Res. Ther. 21, 292 (10.1186/s13075-019-2073-x) 31847895 PMC6918687

[37] Liu Z, Kong F, Vallance JE, Harmel-Laws E, Amarachintha S, Steinbrecher KA, Rosen MJ, Bhattacharyya S 2017 Activation of TGF-beta activated kinase 1 promotes colon mucosal pathogenesis in inflammatory bowel disease. Physiol. Rep. 5, e13181 (10.14814/phy2.13181) 28373409 PMC5392505

[38] Kim SI, Choi ME 2012 TGF-beta-activated kinase-1: new insights into the mechanism of TGF-beta signaling and kidney disease. Kidney Res. Clin. Pract. 31, 94–105. (10.1016/j.krcp.2012.04.322) 26889415 PMC4715161

[39] Wu H, Zhou J, Ou W, Li Y, Liu M, Yang C 2017 TAK1 as the mediator in the protective effect of propofol on renal interstitial fibrosis induced by ischemia/reperfusion injury. Eur. J. Pharmacol. 811, 134–140. (10.1016/j.ejphar.2017.06.009) 28603043

[40] Omori E, Matsumoto K, Sanjo H, Sato S, Akira S, Smart RC, Ninomiya-Tsuji J 2006 TAK1 is a master regulator of epidermal homeostasis involving skin inflammation and apoptosis. J. Biol. Chem. 281, 19 610–19 617. (10.1074/jbc.M603384200) PMC1797070 16675448

[41] Omori E, Morioka S, Matsumoto K, Ninomiya-Tsuji J 2008 TAK1 regulates reactive oxygen species and cell death in keratinocytes, which is essential for skin integrity. J. Biol. Chem. 283, 26 161–26 168. (10.1074/jbc.M804513200) PMC2533783 18606807

[42] Sayama K et al. 2006 Transforming growth factor-beta-activated kinase 1 is essential for differentiation and the prevention of apoptosis in epidermis. J. Biol. Chem. 281, 22 013–22 020. (10.1074/jbc.M601065200) 16754690

[43] Goldmann T et al. 2013 A new type of microglia gene targeting shows TAK1 to be pivotal in CNS autoimmune inflammation. Nat. Neurosci. 16, 1618–1626. (10.1038/nn.3531) 24077561

[44] Katsura H et al. 2008 Transforming growth factor-activated kinase 1 induced in spinal astrocytes contributes to mechanical hypersensitivity after nerve injury. Glia 56, 723–733. (10.1002/glia.20648) 18293403

[45] Pap T, Korb-Pap A 2015 Cartilage damage in osteoarthritis and rheumatoid arthritis--two unequal siblings. Nat. Rev. Rheumatol. 11, 606–615. (10.1038/nrrheum.2015.95) 26195338

[46] Singh AK, Umar S, Riegsecker S, Chourasia M, Ahmed S 2016 Regulation of transforming growth factor beta-activated kinase activation by epigallocatechin-3-gallate in rheumatoid arthritis synovial fibroblasts: suppression of K(63) -linked autoubiquitination of tumor necrosis factor receptor-associated factor 6. Arthritis Rheumatol. 68, 347–358. (10.1002/art.39447) 26473505 PMC5383419

[47] Onuora S 2017 Rheumatoid arthritis: JAK-ing up inadequate RA therapy. Nat. Rev. Rheumatol. 13, 513 (10.1038/nrrheum.2017.114) 28680135

[48] Sato S, Sanjo H, Takeda K, Ninomiya-Tsuji J, Yamamoto M, Kawai T, Matsumoto K, Takeuchi O, Akira S 2005 Essential function for the kinase TAK1 in innate and adaptive immune responses. Nat. Immunol. 6, 1087–1095. (10.1038/ni1255) 16186825

[49] Sanjo H, Takeda K, Tsujimura T, Ninomiya-Tsuji J, Matsumoto K, Akira S 2003 TAB2 is essential for prevention of apoptosis in fetal liver but not for interleukin-1 signaling. Mol. Cell Biol. 23, 1231–1238. (10.1128/MCB.23.4.1231-1238.2003) 12556483 PMC141141

[50] Komatsu Y et al. 2002 Targeted disruption of the Tab1 gene causes embryonic lethality and defects in cardiovascular and lung morphogenesis. Mech. Dev. 119, 239–249. (10.1016/S0925-4773(02)00391-X) 12464436

[51] Tang M et al. 2008 TAK1 is required for the survival of hematopoietic cells and hepatocytes in mice. J. Exp. Med. 205, 1611–1619. (10.1084/jem.20080297) 18573910 PMC2442639

[52] Bettermann K et al. 2010 TAK1 suppresses a NEMO-dependent but NF-κB-independent pathway to liver cancer. Cancer Cell 17, 481–496. (10.1016/j.ccr.2010.03.021) 20478530

[53] Kajino-Sakamoto R, Omori E, Nighot PK, Blikslager AT, Matsumoto K, Ninomiya-Tsuji J 2010 TGF-β-activated kinase 1 signaling maintains intestinal integrity by preventing accumulation of reactive oxygen species in the intestinal epithelium. J. Immunol. 185, 4729–4737. (10.4049/jimmunol.0903587) 20855879 PMC3064262

[54] Le Goff C et al. 2016 Heterozygous mutations in MAP3K7, encoding TGF-β-activated kinase 1, cause cardiospondylocarpofacial syndrome. Am. J. Hum. Genet. 99, 407–413. (10.1016/j.ajhg.2016.06.005) 27426734 PMC4974068

[55] Wade EM et al. 2016 Mutations in MAP3K7 that alter the activity of the TAK1 signaling complex cause frontometaphyseal dysplasia. Am. J. Hum. Genet. 99, 392–406. (10.1016/j.ajhg.2016.05.024) 27426733 PMC4974064

[56] Sakamoto K, Wehde BL, Radler PD, Triplett AA, Wagner KU 2016 Generation of Janus kinase 1 (JAK1) conditional knockout mice. Genesis 54, 582–588. (10.1002/dvg.22982) 27671227 PMC6988131

[57] Conzelmann M et al. 2010 IFN-gamma activated JAK1 shifts CD40-induced cytokine profiles in human antigen-presenting cells toward high IL-12p70 and low IL-10 production. Biochem. Pharmacol. 80, 2074–2086. (10.1016/j.bcp.2010.07.040) 20709027

[58] Sun C, Bernards R 2014 Feedback and redundancy in receptor tyrosine kinase signaling: relevance to cancer therapies. Trends Biochem. Sci. 39, 465–474. (10.1016/j.tibs.2014.08.010) 25239057

[59] Muller J, Ory S, Copeland T, Piwnica-Worms H, Morrison DK 2001 C-TAK1 regulates Ras signaling by phosphorylating the MAPK scaffold, KSR1. Mol. Cell 8, 983–993. (10.1016/S1097-2765(01)00383-5) 11741534

[60] Wu J et al. 2013 Mechanism and *in vitro* pharmacology of TAK1 inhibition by (5Z)-7-oxozeaenol. ACS Chem. Biol. 8, 643–650. (10.1021/cb3005897) 23272696

[61] Kilty I, Jones LH 2015 TAK1 selective inhibition: state of the art and future opportunities. Future Med. Chem. 7, 23–33. (10.4155/fmc.14.138) 25582331

[62] Kamiyama H, Usui T, Sakurai H, Shoji M, Hayashi Y, Kakeya H, Osada H 2008 Epoxyquinol B, a naturally occurring pentaketide dimer, inhibits NF-κB signaling by crosslinking TAK1. Biosci. Biotechnol. Biochem. 72, 1894–1900. (10.1271/bbb.80142) 18603781

[63] Buglio D, Palakurthi S, Byth K, Vega F, Toader D, Saeh J, Neelapu SS, Younes A 2012 Essential role of TAK1 in regulating mantle cell lymphoma survival. Blood 120, 347–355. (10.1182/blood-2011-07-369397) 22649101 PMC3460632

[64] Hornberger KR et al. 2013 Discovery of 7-aminofuro[2,3-c]pyridine inhibitors of TAK1: optimization of kinase selectivity and pharmacokinetics. Bioorg. Med. Chem. Lett. 23, 4511–4516. (10.1016/j.bmcl.2013.06.054) 23856049

[65] Hornberger KR et al. 2013 Discovery and optimization of 7-aminofuro[2,3-c]pyridine inhibitors of TAK1. Bioorg. Med. Chem. Lett. 23, 4517–4522. (10.1016/j.bmcl.2013.06.053) 23850198

[66] Kilty I et al. 2013 TAK1 inhibition in the DFG-out conformation. Chem. Biol. Drug Des. 82, 500–505. (10.1111/cbdd.12169) 23745990

[67] Tan L et al. 2015 Discovery of type II inhibitors of TGFbeta-activated kinase 1 (TAK1) and mitogen-activated protein kinase kinase kinase kinase 2 (MAP4K2). J. Med. Chem. 58, 183–196. (10.1021/jm500480k) 25075558 PMC4292808

[68] Zhou J, Zheng B, Ji J, Shen F, Min H, Liu B, Wu J, Zhang S 2015 LYTAK1, a novel TAK1 inhibitor, suppresses KRAS mutant colorectal cancer cell growth *in vitro* and *in vivo*. Tumour Biol. 36, 3301–3308. (10.1007/s13277-014-2961-2) 25524577

[69] Tan L et al. 2017 Studies of TAK1-centered polypharmacology with novel covalent TAK1 inhibitors. Bioorg. Med. Chem. 25, 1320–1328. (10.1016/j.bmc.2016.11.034) 28038940 PMC5484535

[70] Camacho R, Staruch MJ, Dasilva C, Koprak S, Sewell T, Salituro G, Dumont FJ 1999 Hypothemycin inhibits the proliferative response and modulates the production of cytokines during T cell activation. Immunopharmacology 44, 255–265. (10.1016/S0162-3109(99)00085-5) 10598882

[71] Zhao A et al. 1999 Resorcylic acid lactones: naturally occurring potent and selective inhibitors of MEK. J. Antibiot. (Tokyo) 52, 1086–1094. (10.7164/antibiotics.52.1086) 10695671

[72] Bosman MC, Schepers H, Jaques J, Brouwers-Vos AZ, Quax WJ, Schuringa JJ, Vellenga E 2014 The TAK1-NF-κB axis as therapeutic target for AML. Blood 124, 3130–3140. (10.1182/blood-2014-04-569780) 25287709

[73] Miura T, Matsuo A, Muraoka T, Ide M, Morikami K, Kamikawa T, Nishihara M, Kashiwagi H 2017 Identification of a selective inhibitor of transforming growth factor beta-activated kinase 1 by biosensor-based screening of focused libraries. Bioorg. Med. Chem. Lett. 27, 1031–1036. (10.1016/j.bmcl.2016.12.064) 28109791

[74] Muraoka T, Ide M, Morikami K, Irie M, Nakamura M, Miura T, Kamikawa T, Nishihara M, Kashiwagi H 2016 Discovery of a potent and highly selective transforming growth factor beta receptor-associated kinase 1 (TAK1) inhibitor by structure based drug design (SBDD). Bioorg. Med. Chem. 24, 4206–4217. (10.1016/j.bmc.2016.07.006) 27448772

